# Chthonic severance: dinosaur eggs of the Mesozoic, the significance of partially buried eggs and contact incubation precursors

**DOI:** 10.1098/rstb.2022.0144

**Published:** 2023-08-28

**Authors:** Jason D. Hogan, David J. Varricchio

**Affiliations:** Department of Earth Sciences, Montana State University, Bozeman, MT 59717, USA

**Keywords:** Pennaraptora, partial egg burial, contact incubation

## Abstract

For most dinosaurs, clutches consisted of a single layer of spherical to sub-spherical, highly porous eggs that were probably fully buried. Both eggs and clutch form change drastically with pennaraptoran theropods, the clade that includes birds. Here, far less porous, more elongate eggs are arranged with additional complexity, and only partially buried. While partial egg burial seems to be effective for an extremely small group of modern birds, the behaviour's overall rarity complicates our understanding of Mesozoic analogies. Recent experimental examination of pennaraptoran nesting thermodynamics suggests that partial egg burial, combined with contact incubation, may be more efficacious than has been presumed. We propose that nest guarding behaviour by endothermic archosaurs may have led to an indirect form of contact incubation using metabolic energy to affect temperature change in a buried clutch through a barrier of sediment, which in turn may have selected for shallower clutch burial to increasingly benefit from adult-generated energy until partial egg exposure. Once partially exposed, continued selection pressure may have aided a transition to fully subaerial eggs. This hypothesis connects the presence of partially buried dinosaurian clutches with the transition from basal, crocodile-like nesting (buried clutches guarded by adults) to the dominant avian habit of contact incubating fully exposed eggs.

This article is part of the theme issue ‘The evolutionary ecology of nests: a cross-taxon approach’.

## 1. Introduction

Birds are unique among extant vertebrate classes in that all known species lay eggs [1,2]. It has been suggested that avian anatomy or physiology is simply incompatible with live birth [3–5]; however, Blackburn & Evans [1] instead propose that birds already reap many of the benefits of viviparity (e.g. thermoregulatory contact incubation provides a similar thermal benefit as internal egg retention). Whichever the case, the avian adult-nest unit [6] is highly effective and has probably been a significant factor in birds' widespread success. Thermoregulatory contact incubation is a crucial aspect of the adult-nest unit, made possible through a combination of endothermy, nest attendance and subaerial eggs. Crocodilians, the closest living relatives to birds, also attend their nests but are ectothermic and bury their clutches. Deeming [6, p. 6] summarizes the general scientific consensus that, ‘Presumably contact incubation in birds evolved from a habit of burying and guarding eggs’, but among modern archosaurs the virtually universal adherence to one practice or the other creates a binary dichotomy that obfuscates intermediate possibilities. Luckily the incremental changes in anatomy, physiology and behaviour that made this transition possible are evidenced through the fossil record.

At various stages, the aggregation of these evolutionary innovations would result in reproductive habits notably different than what is seen in either crocodilians or birds. Thermoregulatory contact incubation, as initially defined, successfully encapsulates observable behaviour in extant birds, but the concept lacks granularity when it comes to possible precursor incubation practices. Hogan & Varricchio [7] propose differentiation between strong contact incubation (traditional, as seen in modern birds) and two hypothetical antecedents—that of weak contact incubation (contact incubating partially buried eggs, as suggested for oviraptorosaurs and troodontids) and indirect contact incubation (whereby an endothermic adult provides energy to its fully buried clutch through the substrate medium that separates them). These distinctions allow for further specificity when discussing possible incubation practices in the gap between crocodilian and avian habits.

Even if strong contact incubation only evolved within Neornithes [6,8], many of the steps prior to this culmination, such as single-layer clutches and monoautochronic ovulation, are evidenced by the Mesozoic fossil record. A particular novelty emergent in this transition is the practice of partial egg burial. Partial egg burial is quite rare among extant archosaurs but is well documented within Pennaraptora [9–13]. The general scarcity of modern analogues complicates comparative biology, but the prevalence of this practice in the Mesozoic warrants increased discussion regarding implementation, efficacy and evolutionary significance.

Here, we review reproduction-related fossil material from non-avian dinosaurs, tracing several key behavioural, physiological and anatomical changes from a basal archosaurian state towards modern avian habits. Following this review, we discuss partial egg burial in extant and extinct archosaurs. Finally, we hypothesize how weak and indirect contact incubation may connect with partial egg burial to help fill in crucial gaps that persist in our understanding of the evolution of modern avian reproductive practices.

## 2. Mesozoic dinosaur eggs

Our understanding of nest structure and incubation in non-avian dinosaurs comes primarily from the arrangement of eggs within the substrate and eggshell porosity. Only rarely have nesting traces or other sedimentologic evidence been preserved in association with eggs in the fossil record [11,14,15]. Assemblages of eggs are typically interpreted as a clutch and as reflecting the incubation strategy of the dinosaur. Nevertheless, a clutch emplaced within a substrate may be subsequently disturbed by predation, bioturbation, soil processes or even tectonics. For example, some titanosaur sauropod clutches from the Auca Mahuevo locality in Argentina show extensive modification by vertisol development [16]. Consequently, some discretion is needed when interpreting egg assemblages directly as clutches reflecting the original nesting structure. Importantly, porosity provides an independent check on the incubation environment. High porosity allows for increased gas exchange and typifies eggs incubated within a substrate, whereas low porosity reflects above ground, subaerial incubation [17–20].

**Figure 1.**
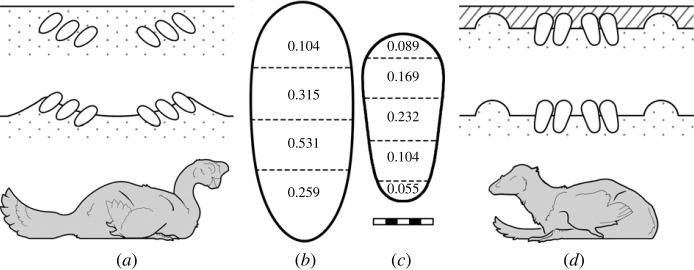
Fossil record evidence of pennaraptoran partial egg burial. (*a*) While many oviraptorosaur clutches have been described, no nest structure has been identified from geological evidence. Nest shape is inferred from clutch arrangement and adult presence. (*b*) Oviraptorosaur eggshell water vapour conductance per cm^2^ (values shown are G_H_2___O/cm^2^_, where G_H_2___O = mg__H_2___O d^−1^_ Torr^−1^), calculated based on eggshell porosity [20]. Variable porosity indicates that the lower portion of the eggs was probably buried while the upper portions were exposed. (*c*) Eggshell water vapour conductance per cm^2^ (G_H_2___O/cm^2^_) was calculated from troodontid eggshell porosity [22]. Similarly heterogeneous porosity also supports partial egg burial in troodontids. (*d*) A troodontid clutch has been described with preserved nest structure in the form of a bowl-shaped depression with a raised rim [11]. A layer of mudstone overlies the clutch and nest-forming micrite. This clear lithologic boundary also supports the notion that the troodontid eggs would have been partially buried in life. Scale bar (5 cm) refers to (*b*) and (*c*).

Taxonomically identified clutches are rare among ornithischians. A clutch for a lambeosaurine hadrosaur consists of 20 large eggs (4190 cm^3^) closely spaced on a single horizon [21]. Those for the hadrosaurine *Maiasaura* are similarly arranged but much smaller [21]. Although reported as occurring in two layers, figured specimens show only one ([21]; figure 1). Norell *et al*. [23] recently described a clutch of *Protoceratops* eggs with embryos. These ellipsoidal, soft-shelled eggs occur more loosely scattered on a single horizon over an area of 1130 cm^2^. High porosity of ornithopod eggs supports subsurface incubation [19,24].

Sauropodomorphs also possessed spherical to sub-spherical eggs ranging from 5 to 7 cm in diameter in the prosauropod *Massospondylus* to 1500 cm^3^ and 4488 cm^3^ in titanosaur sauropod eggs of Argentina and Spain, respectively. *Massospondylus* eggs occur tightly arranged on a single plan and Reisz *et al*. [25] hypothesized that this clutch configuration was primitive among dinosaurs. Titanosaur eggs of the ootaxan *Megaloolithus* show some diversity in their clutch arrangements varying from a single plane, to outlining a depression, or stacked two or three layers deep [26]. Clutches appear to range from 3 to 40 eggs [26]. Clutch sizes may have been constrained by incubation time [27], and large egg counts at Auca Maheuvo may reflect superposition of clutches through soil movement [16]. High porosity of *Megaloolithus* eggs consistently favour incubation within a substrate [19,24], but several clutches from Auca Maheuvo occur within traces of disturbed sediment interpreted as open nests [14]. These traces remain somewhat enigmatic but could represent clutches incubated with vegetation cover or subaerially [14,26]. A number of other dinosaur egg types of more uncertain taxonomic affinity, e.g. *Dendroolithus*, *Dictyoolithus* and *Faveoolithus* share similar sub-spherical to spherical shape, high porosity and largely single-layer clutch configurations [28].

Among theropod dinosaurs, the clade that includes birds, a single-layer clutch occurs within the allosauroid *Lourinhanosaurus* from Portugal. The clutch consists of as many as 100 ellipsoidal eggs, but at approximately 600 cm^3^ in volume each egg is quite small compared to *Lourinhanosaurus*. The eggs are irregularly arranged but closely spaced on a single horizon [29]. By contrast, clutches of pennaraptoran oviraptorosaurs and troodontids show a much more regular structure. Coincident with this shift in clutch morphology occur changes in egg size, shape and ornamentation. For non-avian dinosaurs, pennaraptoran eggs are relatively large in comparison to adult size, but are still far below avian egg mass to body mass ratios. Oviraptorosaur and troodontid eggs are markedly elongated with length width ratios of 2 : 1 to greater than 3 : 1 [30,31]. Egg asymmetry is only pronounced among troodontids, and eggshell microstructure becomes increasingly avian-like as well [8].

Oviraptorosaur clutches commonly consist of rings of paired *Elongatoolithus* eggs two or three eggs deep with the bottom eggs placed closer to the centre [32,33]. Eggs angle down and away from the clutch centre at 35° to 40° degrees [33]. The *Macroelongatoolithus* eggs of large-bodied oviraptorsaurs occur paired in a single large ring [34]. Troodontid clutches consist of 10–24 *Prismatoolithus* eggs steeply inclined to nearly upright within the sediments, their pointed end down. They may angle towards the clutch centre such that the upper blunt ends are in close contact [11]. Several features indicate that portions of the eggs in these pennaraptoran clutches remained exposed during incubation (figure 1): contrasting lithologies surrounding lower versus upper portions of the eggs in *Troodon* [11,35], low porosity compared to other non-avian dinosaurs [19,22,24] and adults in direct contact with portions of the eggs [9,12,13,32,36,37].

Upright eggs implanted within substrates also occur among the Enantiornithes, the dominant bird clade of the Cretaceous. In Argentina and Mongolia, some egg types occur singly with the acute end downwards and blunt face upwards, suggesting that these eggs may have simply been buried in substrates and left [38,39], an incubation mode similar to that of some burrow-nesting megapodes (e.g. maleo, Polynesian scrubfowl and Moluccan megapode) that provide no parental care for their eggs [40,41]. Enantiornithine eggs of the Mongolian *Styloolithus sabathi* occur in small clutches of four to eight elongate and upright eggs [42,43]. Two clutches preserve adult limbs atop the eggs suggesting adult guarding and potentially contact incubation [43]. The latter clutches compare closely to those of troodontids and the ootaxon *Prismatoolithus*.

Overall, most non-pennaraptoran dinosaurs had spherical to sub-spherical eggs, typically arranged tightly to loosely spaced together on a single horizon and buried. By contrast, oviraptorosaurs and troodontids built more structured clutches with their elongate eggs. Adults were then able to sit atop, possibly in contact with some portion of the exposed eggs. More fragmentary specimens suggest that incubation in other non-avian pennaraptorans may have been somewhat different [15,44]. Planted, upright eggs occur within at least some enantiornithines and *Styloolithus* clutches with associated adults suggest similar incubation as in troodontids.

## 3. Efficacy of partial egg burial

Since the basal archosaurian egg state is probably covered [17,19,24,45] and the derived avian egg state is exposed, accepting that behaviour often evolves incrementally means that partial egg burial would be a probable transitional step. In order to proliferate, the behaviour must also have been a viable strategy in and of itself—and partial egg burial does indeed appear to have been common practice among known pennaraptorans (although preservation bias could contribute to its ubiquity; figure 1). Complete egg burial helps keep clutches safe from daily temperature fluctuations, weather and predation. At a cursory glance, it seems that partial burial would weaken these advantages. Eggs are closer to the surface and therefore more susceptible to myriad dangers.

Nevertheless, partial egg burial is exhibited (albeit rarely) among some extant birds, best documented among the Charadriiformes. Maclean [46] highlights approximately 13 species that have been observed deliberately burying (partially or completely) their clutches. Unlike megapodes, which are well known for their fully covered clutches and unusual incubation methods [41,47], Charadriiformes do not warm their clutches via vegetative decay or geothermal energy. Incubation via vegetative decay has been suggested for pennaraptoran dinosaurs [5], but currently no fossil evidence of vegetative debris has been described from pennaraptoran nests.

Grellet-Tinner *et al*. [48] were perhaps the first to compare the nesting practices of Pennaraptora and Charadriiformes, specifically highlighting the partially buried clutches of *Pluvianus aegyptius*. In clutch-burying Charadriiformes, egg burial is often dynamic, with adults altering egg exposure in response to triggers such as predation, intrusion, clutch completion, weather or adult presence/absence [46]. *Pluvianus aegyptius* clutches seem to be always at least partially buried. Contact incubation is required at night (maintaining an average clutch temperature of 37.5°C), but the adults manage this simply by contacting the exposed portions of the eggs [49]. In hot daytime temperatures, *Pl. aegyptius* leaves its clutch, first completely burying it and wets its underbelly at a nearby water source. It then brings this moisture back to moderate clutch or chick temperatures through evaporative cooling [46]. Grellet-Tinner *et al*. [48] suggest that *Troodon formosus* may also have employed thermal inertia and evaporative cooling based on behavioural and environmental similarities. Although it is possible that *T. formosus* engaged in this specialized behaviour, clutch wetting does not consistently accompany partial egg burial even among Charadriiformes. For example, *Peltohyas australis* applies similar attention to its clutch, contact incubating the partially buried eggs in cool weather and further covering them when departing the nest. However, *Pe. australis* does not use evaporative cooling, instead choosing to leave the covered eggs largely unattended in hot conditions [46,50].

Two additional egg-burying charadriiforms are *Charadrius alexandrinus* and *Rhinoptilus cinctus.* Unlike populations of the same species in Europe, *C. alexandrinus* nesting on the Arabian Peninsula consistently orient their eggs sharp end downwards and partially bury them [46,51]. Partially buried *T. formosus* and *Styloolithus* eggs show the same orientation, and while oviraptorosaur eggs are also buried point down they are inclined at approximately 35° [33]—possibly owing to the multi-tiered and sloped nature of the nest. *Rhinoptilus cinctus* eggs are instead oriented horizontally, and unlike most other Charadriiformes the clutch remains partially buried throughout incubation. Adults do not add covering when leaving the nest nor in response to threats [46]. The sediment surrounding the eggs is deliberately compacted by the adult, and curiously it appears that the eggs are never turned during the incubation period [52]. The partially buried and highly organized nature of oviraptorosaur and troodontid nests also seem to preclude egg-turning [11,36]. While this may simply be an expression of more basal physiology, as egg-turning is often harmful in reptiles [53,54], it is notable that an extant bird species seem to successfully contact incubate its partially buried clutch without egg-turning.

For Charadriiformes, Maclean [46] suggests that clutch camouflage enhancement is a major advantage of partial egg burial. Partial burial may also have afforded non-avian dinosaur clutches a measure of concealment, but given egg size, clutch size and egg colouring [20], this seems less effective than in the much smaller (parents, eggs and clutches) and more cryptic Charadriiformes. Maclean [46] also indicates that partial egg burial seems to provide significant thermoregulatory advantages. Egg burial is mostly seen in the tropics and subtropics, and this behaviour appears useful for sheltering clutches from extreme heat—a notion supported by the variable habits of *C. alexandrinus* [46,51]. Furthermore, as seen in some species (e.g. *Pl. aegyptius,* [49]), attending adults are still able to contact incubate clutches via exposed egg surfaces during cooler periods.

Perhaps pennaraptorans were also able to thermoregulate their clutches, providing some measure of moderation for both heat and cold depending on environmental conditions. Contact incubation requires nest attendance and, aside from a few exceptions e.g. Pythonidae, [55], endothermy. Fossil evidence of nest attendance has been described for both troodontids [36] and oviraptorosaurs [9,10,12,13,37], and the behaviour seems highly conserved among archosaurs [45]. There is strong evidence that many dinosaurs were endothermic, especially theropods [56–59]. Specifically for pennaraptorans, evidence from Eagle *et al*. [60] indicates oviraptorosaur body temperatures of 31.9 ± 2.9°C, with more recent oxygen isotope uptake research suggesting body temperatures closer to 35–40°C [61]. Dawson *et al*. [62] estimate a troodontid body temperature range 28–38°C, and histological examination of troodontid material supports an endothermic metabolism [63]. Furthermore, feathers are an important feature of Pennaraptora, hence the name, and were probably widespread in the group [64–69]. Given that these dinosaurs probably exhibited the necessary behaviour (attendance) and physiology (endothermy, insulative integumentary structures) to successfully contact incubation their clutches, the question falls to whether the partially buried clutches themselves could benefit from contact incubation.

While modern Charadriiformes show that contact incubation and partial egg burial can function together in at least some cases, the overall rarity of the behaviour makes it difficult to fully understand associated benefits and drawbacks. Recent actualistic investigation suggests that partially buried eggs might significantly benefit from adult contact [7,70]. In these experiments, a contact incubating surrogate kept partially buried eggs temperatures stable and above ambient air and sediment temperatures, yielding clutch temperatures between crocodilian and avian values even in conditions probably colder than the palaeoenvironments that would have housed the pennaraptoran clutches found in the fossil record [70].

While possibly viable, contact incubation of partially buried eggs was almost certainly less efficacious than contact incubating fully exposed eggs. Energy transfer to substrate would be more pronounced than for a subaerial egg, especially in the often intricately insulated nests of modern birds [71]. Varricchio *et al*. [72], following methods outlined by Erickson *et al*. [73], used lines of von Ebner in embryonic teeth (from partially buried eggs) to estimate the incubation rate for the pennaraptoran *T. formosus*. Their results suggest an incubation period of approximately 74 days for a 314 g egg. This is approximately halfway between what would be expected for a reptilian (107.3 days) or avian (44.4 days) egg of the same mass. Not as efficient as modern birds, but a step in that direction.

## 4. Path to brooding exposed eggs

### (a) Transitions in the fossil record

**Figure 2.**
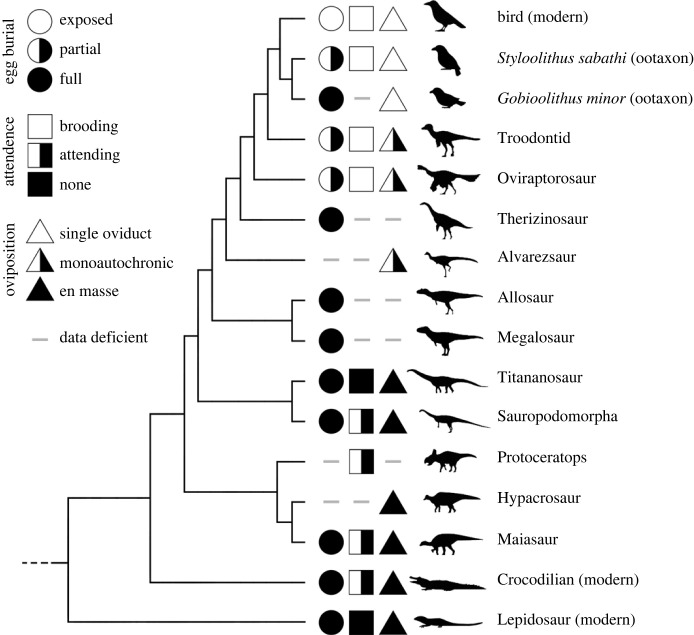
Egg burial, nest attendance and oviposition as evidenced by the fossil record. Many non-avian dinosaurs fully buried their clutches, but partial egg burial is seen among oviraptorosaurs and troodontids. Some enantiornithines appear to exhibit similar behaviour, while others may fully bury their eggs. Fully exposed eggs are currently only known from neornithines. Nest attendance appears highly conserved among archosaurs, excepting titanosaurs. Guarding eventually morphs into contact incubation, possibly in Maniraptora (there is debate over whether oviraptorosaurs and troodontids were capable of contact incubation). En masse egg laying is present in Ornithischia and Sauropodomorpha, giving way to monoautochronic ovulation in maniraptorans and single oviduct physiology in Avialae.

Most modern birds contact incubate a subaerial clutch laid one egg at a time, a habit which probably evolved from guarding buried eggs laid en masse [6,48], behaviour perpetuated to this day by crocodilians. The fossil record provides evidence that much of the necessary anatomical, physiological and behavioural changes required to bridge this gap first evolved within non-avian dinosaurs (figure 2). En masse egg laying is seen in ornithopods [21,74,75] and sauropodomorphs [25,48,76], but along lineages more closely related to Aves (alvarezsaurs, troodontids and oviraptorosaurs), there is evidence of monoautochronic ovulation [8,32,36,37,77]. There is disagreement over whether troodontid clutch arrangements support monoautochronic or single oviduct physiology ([36] versus [48]). It has been proposed that enantiornithine birds had only a single oviduct, but controversy remains here as well [8,78].

Nest attendance is ubiquitous in modern archosaurs and was probably a basal archosaurian practice [19]. Evidence of attendance has been documented for both ornithischian [79,80] and saurischian dinosaurs [9,10,13,25,36]. Closer to Aves, numerous oviraptorosaurs in adult-clutch associations have been found preserved in a ‘brooding’ posture similar to modern birds [9,12,13,37]. However, there is ongoing discussion regarding whether the adults were truly contact incubating [9,13,48,65], guarding [24] or simply caught in the act of oviposition [10,33]—although recent evidence from Bi *et al*. [13] casts doubt on the oviposition hypothesis.

While there has been much discussion concerning non-avian theropods' capacity to contact incubate, there has been far less regarding what led to the change from a subterranean to subaerial clutch in the first place. Traditional contact incubation definitionally requires exposed eggs, and so it is crucial to consider what impetuses might have pushed for subaerial over subterranean clutches. In the archosaurian fossil record, the earliest examples of egg exposure are in the form of partially buried eggs.

### (b) From partial egg burial to full egg exposure

It has been suggested that contact incubating partially buried eggs would be infeasible owing to energy inefficiency and the inability to rotate eggs [81], yet modern Charadriiformes show that contact incubation can indeed be paired with partial egg burial and non-rotated eggs [46,52]. Also, experimental evidence seems to support the possibility that contact incubating partially buried eggs can significantly moderate and elevate clutch temperatures [7,70]. It is unlikely that a behaviour as complex as contact incubation evolved spontaneously without incremental precursors, and it seems prudent to consider such possibilities.

**Figure 3.**
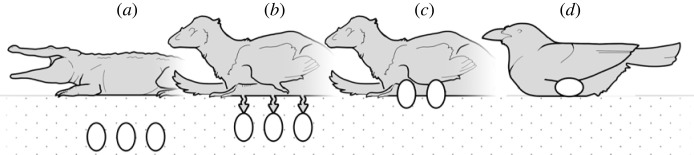
Hypothetical evolutionary path from basal archosaurian to derived avian reproductive practices. (*a*) An ectothermic adult guards a buried clutch laid en masse. This pattern is still used by modern crocodilians. (*b*) An endothermic adult guards a buried clutch. Metabolic energy from the attending adult could perhaps penetrate the substrate and warm its eggs if close enough to the surface in a process of indirect contact incubation. (*c*) An endothermic adult incubates partially buried eggs laid monoautochronically. This is a case of weak contact incubation, where the clutch benefits directly from adult metabolic energy but not to the same degree as fully exposed eggs. (*d*) Strong contact incubation between adult and fully exposed eggs laid one at a time, the method of incubation used by most modern birds.

Given a scenario in which an adult is contact incubating partially buried eggs, there seems to be a clear path to the evolution of fully exposed eggs (figure 3). The more exposed an egg the more it could benefit from a contacting adult's metabolic heat (less sediment contact draining energy). This selective pressure could favour subaerial surface area, and generational iterations would eventually lead to the removal of eggs from the substrate altogether. While this hypothesis may provide a possible explanation for the latter half of the buried to exposed egg trajectory, a driving force selecting towards *partial* burial from *full* burial is less obvious. Nonetheless, extending the above hypothesis may be all that is required.

### (c) From full egg burial to partial egg burial

Fossil evidence indicates that endothermy, along what would become the avian lineage, probably evolved prior to full egg exposure [57,58,82], and since nest attendance is largely conserved in archosaurs, there would probably have been endothermic archosaurs attending their buried clutches. An attending, endothermic adult would warm the substrate beneath it. In most scenarios, this would probably amount to the same as guarding, but perhaps in some cases, the buried clutch was positioned to benefit from adult-generated energy that penetrated the sediment. Even marginal gains in warmth or equilibrium might be significant as temperature is the most important factor in hatching success [83–85]. Clutches laid closer to the surface would benefit more, and so a similar feedback loop as above could select for shallower and shallower egg burial until the clutch is partially exposed. Possibly it was in this manner that the gap from guarded, buried clutches to contact incubated, subaerial clutches was bridged.

Hogan [70] experimentally examines the thermodynamics of oviraptorosaur clutches, with one suite of tests yielding insight into indirect contact incubation. In this experiment, buried emu eggs were raised and kept above ambient temperatures even at depths of 15 cm below the heated surrogate. Predictably, the eggs closest to the surrogate had the highest average temperature at 30.9°C. While significantly higher than experimental ambient air (22.5°C) and ground (19.4°C) temperatures, 30.9°C is still low as an incubation temperature. However, these results should be interpreted alongside several crucial considerations. First, all experimental eggs were infertile. Developing embryos generate heat, raising their own temperatures. Up to a 2.8°C difference has been observed between fertile (37.1°C) and infertile (34.3°C) ostrich eggs [86], and experiments by Ewert & Nelson [87] showed that developing alligator embryos increased clutch temperatures by approximately 0.9°C–3.1°C beyond the sent incubation chamber temperature of 31.8°C.

Second, experimental ambient conditions were probably cool compared to Mesozoic averages [70]. Finally, since the experimental suite was mostly focused on oviraptorosaur nesting, the egg arrangement mimicked the peripheral positioning seen in their clutches. A hypothetical, centrally placed clutch would have experienced greater warming. These tests show that energy can be directed to a clutch through a barrier of sediment, and that energy loss to surrounding sediment does not necessarily outpace input as the eggs readily stayed above ambient temperatures [70]. Notably, factors such as burial depth, ambient temperature, moisture content and substrate composition would all significantly impact efficacy.

In summary, partial egg burial was probably, at some point, an intermediate state between subterranean and subaerial eggs. This adds further resolution to what is a confoundingly binary division in modern archosaurs. Nest attendance is part of even basal archosaurian reproductive habits, and once endothermy evolved then perhaps indirect contact incubation encouraged shallower egg burial to the point of partial exposure. From here, direct adult-egg contact further advantaged clutches. This weak contact incubation may have become increasingly effective as eggs became increasingly free from sediment, eventually culminating in the subaerial clutches and strong contact incubation seen in modern birds.

### (d) Limitations

These hypotheses are in part built from data derived from experiments focusing on egg temperatures in buried or partially buried scenarios [7,70]. However, there are certainly factors beyond clutch temperature that would have complicated the transition from buried to subaerial eggs. For example, embryonic respiration is a notable potential bottleneck. If buried or partially buried clutches increasingly benefited from adult-generated heat, the associated acceleration in embryonic development would require increased oxygen intake that may have been hindered by sediment contact. Additionally, eventual adult contact of partially exposed eggs would bring greater warmth but further limit egg surface available for gas exchange.

Perhaps adaptations beyond increased eggshell pore count were needed to overcome this hurdle. Deeming [88] describes a similar issue when considering the low porosity of *Ornitholithus* eggs (only 15–20% of what is seen in comparably sized modern bird eggs). The purpose of egg ornamentation, prevalent among some pennaraptorans, has not been investigated thoroughly, but it is conceivable that such structures facilitated respiration by creating micro-pockets of air around a buried egg [42,48].

Varricchio *et al*. [72] show that troodontid incubation time is still significantly longer than modern contact incubating birds. It seems possible that troodontid incubation times could have been lengthened by slowed embryonic development from lower clutch temperatures or decreased rates of embryonic respiration. It is plausible that temperature, embryonic respiration or other physiological processes all throttled embryonic development at different points along the evolutionary trajectory from buried to exposed eggs. Potential limitations beyond clutch temperature are certainly worth future investigation.

## 5. Conclusion

Most birds contact incubate subaerial clutches, whereas crocodilians guard subterranean or covered clutches. Presumably avian nesting strategies evolved from basal reptilian practices similar to those seem in today's crocodilians, and the Mesozoic fossil record provides evidence of the necessary incremental changes that made this transition possible. Nevertheless, the binary nature of modern archosaur incubation habits complicates our understanding of dinosaurian incubation. Partially buried eggs, perhaps an intermediate state between burial and exposure, seem to become common in pennaraptoran dinosaurs. Although rare, several modern bird species construct partially buried clutches, however the advantages and disadvantages of this nesting style are not well known.

We hypothesize that (at some point in the archosaur lineage) attending, endothermic adults may have been able to warm their fully buried clutches through a sediment barrier, over time selecting for shallower burial. This indirect contact incubation would not be as efficacious as modern avian incubation but was perhaps successful enough to lead to partially exposed eggs. Partially exposed eggs would receive greater thermoregulatory benefits from an attending adult's metabolic energy in a process of weak contact incubation, a behaviour possibly exemplified by oviraptorosaurs and troodontids. Greater egg exposure would increase these benefits, culminating in the strong contact incubation of fully exposed eggs as seen in modern birds. This trajectory provides a possible solution to the gap between modern archosaurian incubation strategies, furthering discussion regarding the pressures that encouraged a shift from subterranean to subaerial incubation in the avian lineage.

## Data Availability

This article has no additional data.
